# Macrophage targeting of nitazoxanide-loaded transethosomal gel in cutaneous leishmaniasis

**DOI:** 10.1098/rsos.220428

**Published:** 2022-10-05

**Authors:** Husna Khalid, Sibgha Batool, Fakhar ud Din, Salman Khan, Gul Majid Khan

**Affiliations:** ^1^ Nanomedicine Research Group, Department of Pharmacy, Quaid-i-Azam University, 45320 Islamabad, Pakistan; ^2^ Department of Pharmacy, Quaid-i-Azam University, 45320 Islamabad, Pakistan; ^3^ Islamia College University, Peshawar, Khyber Pakhtunkhwa, Pakistan

**Keywords:** cutaneous leishmaniasis, nitazoxanide, transethosomes, transethosomal gel, Box-Behnken design, topical delivery

## Abstract

Topical delivery is preferable over systemic delivery for cutaneous leishmaniasis, because of its easy administration, reduced systemic adverse effects and low cost. Nitazoxanide (NTZ) has broad-spectrum activity against various parasites and has the potential to avoid drug resistance developed by enzymatic mutations. NTZ oral formulation is associated with severe dyspepsia and stomach pain. Herein, NTZ-transethosomes (NTZ-TES) were prepared and loaded into chitosan gel (NTZ-TEG) for topical delivery. NTZ-TES were prepared by the thin-film hydration method and optimized statistically via the Box-Behnken method. The optimized formulation indicated excellent particle size (176 nm), polydispersity index (0.093), zeta potential (−26.4 mV) and entrapment efficiency (86%). The transmission electron microscopy analysis showed spherical-sized particles and Fourier-transform infrared spectroscopy analysis indicated no interaction among the excipients. Similarly, NTZ-TEG showed optimal pH, desirable viscosity and good spreadability. NTZ-TES and NTZ-TEG showed prolonged release behaviour and higher skin penetration and deposition in the epidermal/dermal layer of skin in comparison with the NTZ-dispersion. Moreover, NTZ-TES showed higher percentage inhibition, lower half-maximal inhibitory concentration (IC_50_) against promastigotes and higher macrophage uptake. Additionally, skin irritation and histopathology studies indicated the safe and non-irritant behaviour of the NTZ-TEG. The obtained findings suggested the enhanced skin permeation and improved anti-leishmanial effect of NTZ when administered as NTZ-TEG.

## 1. Introduction

Leishmaniasis refers to the complex tropical infectious disease which has been neglected for several decades. There are more than 20 different types of intracellular obligate pathogenic parasites that belong to the genus *Leishmania*, which are responsible for the development of this disease in mammals [1]. Cutaneous leishmaniasis (CL) is a type of leishmaniasis which is manifested by the development of skin lesions or ulcers at the site of a sand fly bite*.* In most cases, this infection is self-healing, but in cases of New World, the infection may be worsened by outspreading to supplementary areas of the body, i.e. lymph nodes or cutaneal sites. Mucous membrane destruction may occur in cases of these severe metastatic lesions, leading to massive deformability or even death of individuals [2].

Currently, a lot of chemotherapeutic agents are used for the treatment of lesihmaniasis including Miltefosine [3] Imiquimod [4], Amphotericin B [5] and Trifluralin [6]; however, they are not very effective [7]. Moreover, the available anti-leishmanial agents are associated with several drawbacks; i.e. toxicity, high cost and drug resistance [8]. Therefore, drug discovery and development programmes are mainly focusing on the development of new drugs which are selective, have a high therapeutic index and are cost effective, especially in developing countries [9]. Furthermore, these drugs are considered as advanced novel therapeutic agents which mainly target the metabolic pathways of parasites or produce reactive oxygen species [10], cause programmed cell death [11], or inhibit ergosterol production [12]. Therefore, such anti-parasitic agents are considered as potential candidates for CL treatment which either produce oxidative stress or inhibit the vital redox reactions inside the parasites [10].

Nitazoxanide [2-(5-nitrothiazol-2-ylcarbamoyl) phenyl acetate; Alinia®] (NTZ) is a broad-spectrum anti-parasitic agent that belongs to a nitro-heterocyclic class named thiazolides [13]. NTZ shows its activity against protozoa by inhibiting the pyruvate : ferredoxin oxidoreductase enzyme-dependent electron transfer reaction, which is needed by protozoans and anaerobic bacteria for their anaerobic energy metabolism [14]. The enzyme pyruvate kinase catalyses the glycolysis process which results in the production of adenosine triphosphate (ATP). By inhibiting ATP production, NTZ may have potential to inhibit the leishmanial parasites [15].

NTZ has potential to avoid the drug resistance developed by enzymatic mutations by inhibiting first step, i.e. causing the pyruvate binding to the cofactor thiamine pyrophosphate in opposition to the binding with potential substrates in the cell [16]. Moreover, it has more specific activity for *Leishmania* as compared to the other second-line anti-parasitic agents being more specific for bacterial or fungal infections [15]. In a previous study, the *in vivo* results indicated that by giving NTZ orally at the dose of 400 mg kg^−1^ d^−1^, the liver and splenic leishmanial parasitic load was reduced by more than 90% [17]. Yet, the prolonged and increased oral dose of NTZ resulted in adverse events i.e. abdominal pain, diarrhoea, headache and nausea [18]. In another study, the NTZ-loaded liposomes, at the dose of 2 mg kg^−1^ d^−1^ intraperitoneally, indicated 82% and 50% reduction in liver and splenic parasitic load, respectively [19]. The *in vitro* activity of NTZ has been reported against *Leishmania mexicana, Plasmodium berghei*, *Trypanosoma curzi*, *Entamoeba histolytica* and *Trichomonas vaginalis* [20,21], while *in vivo* therapeutic activity of NTZ in BALB/c mice against *Leishmania donovani* was first reported by Zhang *et al*. [17]. Recently nano-liposomal formulations of NTZ were developed and tested against *Leishmania infantum,* causing visceral leishmaniasis, which showed good *in vivo* efficacy at low doses, thus have potential as a new anti-leishmanial agent [19]. In our study, we developed the topical transethosomal formulation of NTZ (containing 4.3 mg of NTZ per 2 g of the gel), which mainly targets the CL, by deeper penetration and enhanced deposition of drug to the target sites.

**Figure 1.**
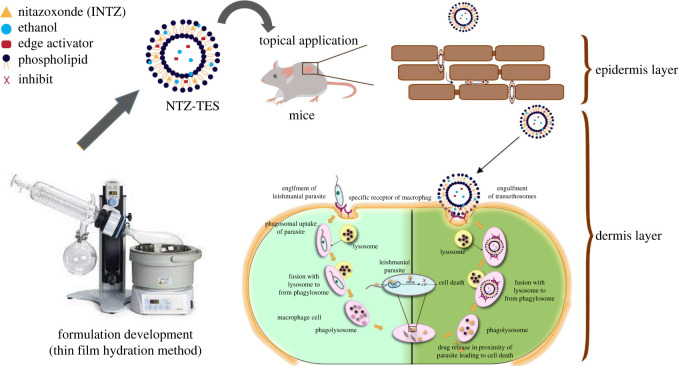
Illustration of the preparation of NTZ-TES and their mechanism of targeting *Leishmania*-effected macrophages.

Although topical drug delivery system has various advantages the conventional systems i.e. creams, ointments and gels cannot penetrate into the deeper skin layers [22,23]. Thus, nano-sized novel drug delivery systems, i.e. lipid-based ultra-deformable nano-vesicles, are preferable for deeper skin penetration [24,25]. Transethosomes (TES) are advanced ultra-deformable vesicles having characteristics of both transfersomes and ethosomes [26]. They are more preferable than transferosomes in the case of hydrophobic drugs, because loading into transfersomes becomes difficult as these drugs can interact with the lipid bilayers, resulting in the deformability of these vesicles and loss of elasticity [27]. The low dose of drug entrapped in nano-carriers when applied topically not only saves the patient from painful invasive parenteral therapy, but also avoids the systemic adverse effects produced by the oral or parenteral route. TES alone have low viscosity, making it difficult to retain on the desired site of action for extensive periods of time. Therefore, NTZ-TES were loaded into chitosan gel for appropriate topical drug delivery [28]. Chitosan was used as a gel base for the preparation of nitazoxanide-loaded transethosomal gel (NTZ-TEG) owing to its non-toxic nature, greater stability and wound healing properties [29]. In this study, we reported the fabrication of NTZ-TES topical formulation with significantly increased dermal penetration, reduced cytotoxicity and enhanced anti-leishmanial efficacy (figure 1). To the best of our knowledge, no such study has been reported so far. Moreover, the incorporation of NTZ in TEG for CL has not been reported earlier.

## 2. Experimental

### 2.1. Materials

NTZ was purchased from Macklin Biochemical Co., Ltd (Shanghai, China). Phospholipon 90 G (PL90G) was a generous gift donated by Lipoid AG, Switzerland. Roswell Park memorial institute (RPMI-1640) medium, Medium-199 (M-199), fetal bovine serum (FBS) and penicillin/streptomycin (10 000 U ml^−1^) were obtained from Thermo Fisher Scientific, USA. All other reagents used were of pure analytical grade.

### 2.2. Methods

#### 2.2.1. Preparation of transethosomes

The TES were prepared by the thin-film hydration method described by Garg *et al.* with a few modifications [30]. For the preparation of blank TES, the specified amounts of PL90G and Span 80 (S80) were added in 1 : 1 v/v blend of organic solvents i.e. chloroform and methanol and placed in a rotary flask. A rotary evaporator was used to remove the organic solvents at 50°C and 100 rpm. The hydration of deposited thin film was performed by using 10 ml of ethanolic-phosphate buffered saline (PBS) (containing 20–40% (v/v) ethanol), at 60°C and 100 rpm for 1 h. The obtained vesicles were then kept aside for another half hour to hydrate completely and then subjected to a size reduction process by passing through 0.22 and 0.45 nm polycarbonate membrane syringe filters [31].

Similarly for the preparation of NTZ-TES, the NTZ was added in methanol and sonicated for 5 min, while PL90G and S80 were added to chloroform [19]. Both the solutions were then combined in a round-bottom flask and organic solvents were then removed by rotary evaporator under reduced pressure as shown in the electronic supplementary material, figure S1. The rest of the procedure was same as for the preparation of blank TES [30].

#### 2.2.2. Preparation of nitazoxanide-loaded transethosomal gel

In order to make the NTZ-TES rheologically acceptable for onsite application, they were incorporated into (2% w/v) chitosan gel. For 3 ml preparation of NTZ-TEG, 100 mg of low- or medium-molecular weight chitosan was dissolved in 1% (v/v) acetic acid solution with continuous stirring and then 5 ml final volume was made up by adding 2 ml of PBS 7.4 having optimized NTZ-TES pellets dispersed in it. The mixture was stirred continuously until the formation of a homogeneous gel [32].

#### 2.2.3. Experimental design for optimization of blank transethosomes

For the optimization of blank TES, a two-level three-factors, Box-Behnken Design was used by employing the software Design Expert® (Version 12, Stat-Ease Inc., USA). The independent variables selected were lipid, surfactant and ethanol while dependent variables were particle size (PS), polydispersity index (PDI) and zeta potential (ZP) [31].

### 2.3. Characterization studies

#### 2.3.1. Nitazoxanide-transethosomes, particle size, polydispersity index, zeta potential determination and transmission electron microscopy analysis

The dynamic light scattering method was employed to characterize NTZ-TES in terms of PS, PDI and ZP using the Zetasizer (Malvern Zetasizer, ZS 90). Each measurement was recorded in triplicate (mean ± s.d.). The scanning was performed at 25°C and 90° scattering angle. ZP was determined by the use of universal-dip-cell in the Zetasizer ZS 90 [33,34].

The superficial morphology of NTZ-TES was examined by using transmission electron microscopy (TEM). In this analysis, the prepared NTZ-TES formulation was placed on a grid of copper coated with carbon in droplet form and allowed to stand for some time so that it got stuck to the grid. After that a 1% phosphotungstic acid was used as the negative stain and TEM analysis was then performed at 100 kV [34–36].

#### 2.3.2. Entrapment efficiency

The indirect method was employed to determine the entrapment efficiency (%EE) of TES. The prepared NTZ-TES were subjected to centrifugation at 15 000 rpm for the period of 2 h. After the centrifugation, the supernatant was separated from the pellets, diluted with acetonitrile and then UV-visible spectrophotometric analysis was done to measure free NTZ. The following equation was then employed for the calculation of %EE [32,34]):

$$\text{\%}\;\mathrm{EE}=\frac{\mathrm{total}\;\mathrm{amount}\;\mathrm{of}\;\mathrm{drug}-\mathrm{unentraped}\;\mathrm{drug}}{\mathrm{total}\;\mathrm{amount}\;\mathrm{of}\;\mathrm{drug}}\times 100.$$

#### 2.3.3. Compatibility study of drug and excipients

In order to determine the compatibility of drug and excipients, Fourier-transform infrared spectroscopy (FTIR) analysis of the pure NTZ, physical mixture (PM), PL90G, and optimized NTZ-TES was performed. About 10 mg of sample was compressed along with 200 mg of KBr into a disc using a hydraulic press and FTIR spectra was measured over the range of 500–4000 cm^−1^ using an FTIR spectrophotometer [37,38].

#### 2.3.4. Physico-chemical and rheological study of nitazoxanide-loaded transethosomal gel

The colour, appearance and homogeneity of both the blank and NTZ-TEG was evaluated visually [39]. A digital pH-meter was used for the measurement of gel pH, for which 100 mg of the optimized gel was added to the 50 ml of distilled water and stirred to dissolve completely [40]. The viscosity of the both the blank and drug-loaded gel was determined by using a Brookfield cone and plate rheometer at room temperature using spindle CPA 52 Z [41,42].

#### 2.3.5. Spreadability measurement

For measurement of gel spreadability, an area of 2 cm was marked on the glass slide and gel of 500 mg was placed. Then, one more glass slide along with a 500 g weight was placed on first glass slide having gel on it for 5 min at a room temperature to allow proper spreading of the gel. After that, the increase in area of gel spread was measured and the percentage spreadability of gel was calculated by the following equation [43]:

$$\text{\%}\;\mathrm{spread}\;\mathrm{area}=\frac{\mathrm{final}\;\mathrm{area}\;\mathrm{after}\;\mathrm{spreading}}{2\;\mathrm{cm}}\times 100.$$

#### 2.3.6. *In vitro* release and kinetic drug release study

For investigation of the NTZ release behaviour, an *in vitro* study was performed for NTZ-dispersion, NTZ-TES and NTZ-TEG by using the PBS of pH 5.5 as the release medium. pH 5.5 depicts the pH of the macrophage endosomes and also the pH of skin [44]. The NTZ-dispersion, NTZ-TES and NTZ-TEG having equivalent amount of drug were added into the dialysis bag (MWCO, 12–14 kDa). These dialysis bags were then placed in water-bath-shaker having release media with a controlled temperature of 37 ± 0.5°C. The samples after a predetermined time were taken out and replaced with the fresh PBS solution to maintain sink conditions. The drug concentration in the samples was determined by using a UV spectrophotometer at 335 nm in triplicate after diluting the samples with a predetermined ratio of acetonitrile. The cumulative amount of the drug released was then calculated by using the following formula [45]:

$$\mathrm{Qn}=Vr*Cn+\overset{n-1}{\underset{i=0}{\sum }}.Vs*Ci.$$

#### 2.3.7. *Ex vivo* permeation and skin deposition study

For the *ex vivo* permeation study, albino Wister rats (weight 100–125 g) were purchased from the Central Animal House of NIH, Islamabad, and were sacrificed. Their skin was isolated and used in this study. Moreover, the *ex vivo* permeation study was executed using a nine stations Franz diffusion cell apparatus (Premerger, PA, USA). The rat skin with 400 µm ± 20 µm thickness was excised and clipped between the donor and receiver compartment with upwards facing the stratum corneum (SC). In the receiver compartment, the PBS (pH 7.4) was filled and stirring at 300 rpm was kept fixed. The NTZ-dispersion, NTZ-TES and NTZ-TEG were then placed in the donor compartment and a 24 h study was performed. The temperature of 32 ± 1°C was maintained throughout the experiment to simulate the skin conditions. The samples were then withdrawn at predetermined times (0, 0.25, 0.5, 1, 2, 4, 6, 12 and 24 h) and analysed for NTZ using a UV spectrophotometer [46,47].

The steady state flux (Jss), the enhancement ratio (ER) and permeability coefficient (Kp) were determined by employing following equations [48]:

$$\mathrm{Jss}=\frac{\mathrm{amount}\;\mathrm{of}\;\mathrm{permeated}\;\mathrm{drug}\;}{\mathrm{time}},$$

$$\mathrm{ER}=\frac{\mathrm{Jss}\;\mathrm{of}\;\mathrm{the}\;\mathrm{formulation}}{\mathrm{Jss}\;\mathrm{of}\;\mathrm{the}\;\mathrm{pure}\;\mathrm{drug}\;\mathrm{dispersion}},$$

$$\mathrm{and}\;\mathrm{Kp}=\frac{\mathrm{Jss}}{\mathrm{initial}\;\mathrm{amount}\;\mathrm{of}\;\mathrm{NTZ}\;\mathrm{in}\;\mathrm{donor}\;\mathrm{cell}}.$$

After completion of the 24 h study, the samples of skin were removed carefully, washed with PBS and dried by blotting to remove excess formulation. The skin was chopped into small fragments, homogenized and then sonicated in 20 ml acetonitrile for 1 h to extract the drug completely. After that the sample was centrifuged at 10 000 rpm for 15 min, and the supernatant was taken to estimate the amount of drug deposited in SC and dermal layers by using a UV-spectrophotometer [49].

#### 2.3.8. Evaluation of skin structure after treatment

Any change in the lipid organization of the epidermal skin layer produced by the permeation enhancers after treatment with the NTZ-dispersion and NTZ-TEG was evaluated by FTIR [50]. The epidermal layer was separated from the dermal layer by using isopropyl alcohol and placed in distilled water at 60°C for 2 min. The isolated epidermal layer was placed in a Franz diffusion cell with NTZ-dispersion and NTZ-TEG applied on the skin for the period of 6 h under same conditions as used for the permeation study. After 6 h, the epidermis was removed from the diffusion cell, washed with PBS and dried properly. This dried epidermis was used to evaluate the functional groups of the lipids under FTIR at the wavenumber of 4000–650 cm^−1^ [51,52].

#### 2.3.9. *In vivo* histopathology and skin irritation study

A skin irritation experiment was carried out according to the procedure given by Draize *et al*.[53] The animals were divided into four groups (*n* = 4) and skin was shaved. One group was treated with NTZ-TEG, a second group with 0.8% formalin and a third group was kept untreated. The skin of the animals was examined after topical application of NTZ-TEG and formalin (0.8%). The topical irritation, i.e. edema or erythema was scored and calculated according to the Draize scoring method [53]. The primary dermal irritation index (PDII) was obtained by the addition of scores of erythema and edema for each study group. According to the PDII, a score of less than 2 indicates non-irritant behaviour, 2–5 indicates irritation and greater than 5 shows the highly irritant nature. After 72 h the animals were sacrificed and histopathological evaluation was performed for each group [54]. The study was conducted as per the approved procedures of National Institute of Health (NIH) and ARRIVE guidelines. Ethical approval of the animal study was granted by the Bioethical Committee of Quaid-i-Azam University Islamabad under the approval number BEC-FBS-QAU-2020-240.

#### 2.3.10. *In vitro* leishmanicidal activity against promastigotes

*In vitro* anti-leishmanial activity was carried out by using diphenyl-tetrazolium (MTT) colorimetric assay. The parasites were incubated in the M-199 medium having 1% FBS, 100 IU ml^−1^ penicillin and 100 µg ml^−1^ streptomycin-sulfates at 24°C. The promastigotes were counted in a Neubauer hemocytometer and were seeded at the concentration of 1 × 10^6^ promastigotes ml^−1^ in each well of a 96-well plate having 20 µl samples, at the concentration of 100 µg ml^−1^ (having less than 1% dimethyl sulfoxide (DMSO) in PBS). DMSO (1%) and amphotericin B (10 µg ml^−1^) in PBS were employed as negative and positive controls, respectively. Incubation of these culture plates was done for the period of 72 h at a temperature of 24°C. After this the sterilized, pre-filtered MTT solution at a concentration of 4 mg ml^−1^ in distilled water was prepared, added to the culture plates and incubated for 4 h at 24°C. After that, the supernatant was carefully removed without disturbing the sediment having formazan crystals. For the dissolution of formazan crystals, DMSO (100 µl) was added in the sediment. After 1 h, the absorbance was measured at the wavelength of 540 nm using the microplate reader. The half-maximal inhibitory concentration (IC_50_) was calculated by using GraphPad prism software [55]. The percentage growth inhibition was then calculated by following equation [56]:

$$\mathrm{growth}\;\mathrm{inhibition}\;(\text{\%})=\frac{\mathrm{optical}\;\mathrm{density}\;\mathrm{of}\;\mathrm{control}-\mathrm{optical}\;\mathrm{density}\;\mathrm{of}\;\mathrm{test}}{\mathrm{optical}\;\mathrm{density}\;\mathrm{of}\;\mathrm{control}}\times 100.$$

#### 2.3.11. *Ex vivo* cell uptake study

The resident macrophages were collected from the peritoneal cavity of the rats by injecting the cold RPMI (cRPMI) media. Moreover, the media containing these macrophages was withdrawn slowly and placed on ice. The collected suspension was centrifuged at 4°C for 10 min. The obtained pellets were then dispersed in the cRPMI media containing 10% FBS and 3% penicillin/streptomycin. The cells were counted using a hemocytometer and placed in the 96-well plate at the concentration of 2 × 10^4^ million cells per well and incubated for 24 h with 5% CO_2_ supply at 37°C [57].

For the cell uptake experiment, the harvested macrophages were employed. The macrophages were washed with sterile PBS to remove the non-adherent cells. After that, NTZ-dispersion and NTZ-TES at the concentration of 50 µg ml^−1^ were added into a 96-well plate and incubated for 24 h in a controlled environment of 5% CO_2_ and at 37°C. After 24 h, the supernatant was removed, and macrophages were washed twice with sterile PBS to remove the free drug. The attached macrophages were scraped off and centrifugation of cellular suspension was carried out at 4°C for 5 min to obtain pellets of macrophages. Methanol was added and sonicated for 5 min. The UV- spectroscopic examination was done to determine the NTZ content up taken by the macrophages [44].

#### 2.3.12. Stability study

The stability study was conducted at temperatures 4 ± 2°C and 30 ± 2°C for a period of four months to evaluate the effects of different storage conditions on the optimized formulation. Samples stored at respective temperatures were evaluated for PS, PDI, ZP and the physical stability [30,58].

## 3. Results and discussion

### 3.1. Optimization and analysis of transethosomes by Box-Behnken design model

For the optimization of blank TES, a two-level, three-factors Box-Behnken design was used by employing the software Design Expert® (version 12, Stat-Ease Inc., USA). The Box-Behnken design was applied specifically because of lesser runs generated than a central composite design, analysing three to four variables. It is preferred if the goal is to accurately optimize a process using a minimum number of experiments in less time and thus provide a far more efficient and cost-effective technique than the conventional processes of formulating and optimization of dosage forms [59]. In table 1, the chosen independent and dependent variables along with observed responses are shown. The levels of independent variables and constraints of dependent variables in the Box-Behnken design are given in the electronic supplementary material, table S1.

**Table 1.** Observed responses for optimization of blank TES. (All the values here represent mean ± s.d. PS, particle size; PDI, polydispersity index; ZP, zeta potential; mg, milligram; nm, nanometer; mV, millivolt.)

independent variables				dependent variables		
formulation codes	phospholipid (mg)	Span 80	ethanol (%)	PS (nm)	PDI	ZP (mV)
TES-1	85	10	40	154.1 ± 3.33	0.093 ± 0.008	−36.23 ± 1.07
TES-2	95	15	30	172.0 ± 2.97	0.137 ± 0.018	−22.20 ± 1.82
TES-3	90	15	20	184.5 ± 2.15	0.184 ± 0.024	−15.63 ± 1.36
TES-4	95	5	30	164.6 ± 1.71	0.119 ± 0.046	−31.03 ± 2.41
TES-5	85	10	20	188.5 ± 3.08	0.138 ± 0.024	−15.40 ± 2.56
TES-6	90	5	20	188.8 ± 1.37	0.142 ± 0.009	−18.03 ± 2.84
TES-7	85	15	30	157.5 ± 2.25	0.106 ± 0.034	−19.06 ± 0.85
TES-8	95	10	20	186.5 ± 4.89	0.172 ± 0.024	−18.10 ± 1.45
TES-9	90	5	40	132.3 ± 1.05	0.077 ± 0.037	−44.73 ± 1.42
TES-10	90	15	40	135.9 ± 1.04	0.075 ± 0.012	−44.23 ± 4.84
TES-11	85	5	30	167.0 ± 3.25	0.091 ± 0.036	−22.00 ± 0.86
TES-12	95	10	40	126.2 ± 0.49	0.105 ± 0.005	−46.00 ± 2.30

The regression analysis for all the responses i.e. PS, PDI and ZP was done by the software shown in the electronic supplementary material, table S2. The obtained results from regression analysis indicated that the models produced for each response were significant with *p* < 0.05. ‘Adequate precision’ was calculated for each response in order to confirm that the given model can navigate the design space and it was observed that the signal to noise ratio was adequate as all the values were greater than 4. Moreover, ‘predicted *R*^2’^ values and ‘adjusted *R*^2’^ values were reasonable in agreement for all the responses, i.e. difference is less than 0.2 [60]. The magnitude of the effect of each factor was determined by the coded equations generated by the software. A positive value represents the synergic effect of the factor on the response while an antagonistic effect is represented by negative value:

$$\mathrm{PS}:R_{1}=+163.17-2.24A-0.3458B-24.97C,$$

$$\mathrm{PDI}:R_{2}=+0.1202+0.0133A+0.0093B-0.0358C,$$

$$\mathrm{and}\;\mathrm{ZP}:R_{3}=-27.72-3.08A+1.83B-13.00C.$$

### 3.2. Effect of the independent variables on particle size

**Figure 2.**
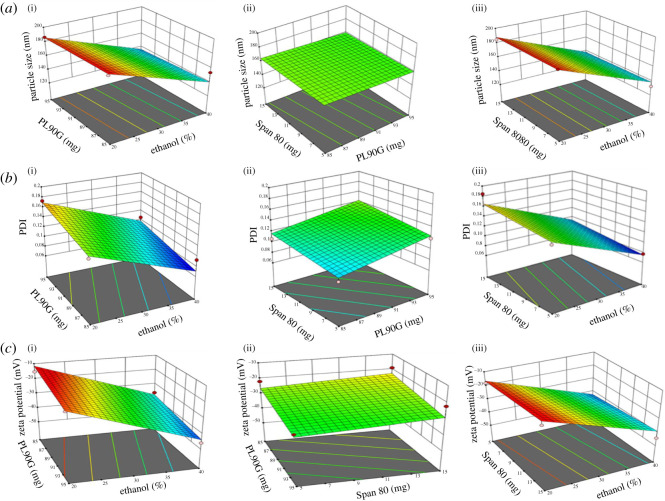
Response-surface three-dimensional graphs indicating effect of the independent variables on (*a*) PS, (i) effect of ethanol and lipid, (ii) effect of lipid and surfactant, and (iii) effect of ethanol and surfactant; (*b*) polydispersity index, (i) effect of ethanol and lipid, (ii) effect of lipid and surfactant, and (iii) effect of ethanol and surfactant; (*c*) ZP, (i) effect of ethanol and lipid, (ii) effect of lipid and surfactant, and (iii) effect of ethanol and surfactant. In figure, particle size, PDI and zeta potential, respectively, represent PS, polydispersity index and ZP.

The size of a particle is crucial in terms of TES permeation from tiny skin pores. The smaller size i.e. less than 200 nm is desirable for the effortless permeation of TES from skin. The size of all prepared TES was within the desirable range i.e. ranging from 126.16 to 188.8 nm. Design Expert® was employed for the data analysis of every response and ANOVA was employed to estimate the effect of each factor on all responses along with the *p*-values. *p* < 0.05 indicates results that are significant. The effect of independent variables i.e. PL90, S80 and ethanol on PS of the TES is shown in figure 2*a*. The three-dimensional plots showed that by changing the ethanol concentration there was a significant (*p* < 0.0001) effect on the PS, and it produced a negative effect on PS, i.e. by increasing the ethanol concentration there was a significant reduction in PS of TES. The reason behind this reduction was that the increased ethanol concentration causes the thinning of the vesicle membrane owing to interpenetration of hydrocarbon chains of ethanol within the lipid bilayers [61]. Moreover, ethanol imparts a negative charge over the vesicular system resulting in reduced mean PS owing to steric stabilization [62]. On the other hand by increasing the surfactant concentration, a non-significant (*p* = 0.9083) reduction in PS was observed which might be owing to the softening effect of surfactant on membrane and owing to the increased elasticity of vesicular membrane [63,64]. Moreover, the increased surfactant concentration could cause the reduction in interfacial tension between lipid and the external medium resulting in smaller PS [65]. Similarly, a non-significant (*p* = 0.4633) PS reduction was observed by increasing the lipid concentration, which has also been reported by previous studies. This reduction was owing to the fact that at higher concentration of lipid, more phospholipids are available which might act as a surfactant thus increasing the stabilization of vesicles and favour the formation of smaller particles [66,67].

### 3.3. Effect of independent variables on polydispersity index

PDI indicates the homogeneity of the formulation. The higher PDI indicates the heterogeneous PS of the nanoparticles which in turn may affect the stability of the formulation. The PDI of the blank TES ranges from 0.075 to 0.185 which is within the optimal range.

The three-dimensional graphs shown in figure 2*b* indicated that there was a negative correlation between the ethanol and PDI. By increasing the ethanol concentration, a significant (*p* < 0.001) reduction in PDI was observed. Similar results were reported previously by Nayak *et al*. [68]. This is because the ethanol imparts negative charge over the vesicular system resulting in reduced mean PS and PDI of TES owing to steric stabilization [62]. The effect of surfactant on PDI was positive, i.e. by increasing the surfactant a non-significant (*p* = 0.0952) increase in PDI was observed, the same effect was also reported in a previous study [69]. A significant (*p* = 0.026) increase in PDI was observed by the increasing lipid concentration which was probably owing to the increase in number of the non-uniform size vesicles formed as a result of increased lipid concentration [70].

### 3.4. Effect of independent variables on zeta potential

For the long-term stability of nanoparticles, ZP values must be in optimal range. The particles with ZP more than ±60 mV have excellent stability properties while less than ±10 mV indicates poor stability properties resulting in rapid agglomeration of the nanoparticles. The ZP of the blank TES ranges from −45 to −15.4 mV which is in the optimal range.

The three-dimensional plots shown in figure 2*c* indicated that by increasing the ethanol concentration the ZP of TES significantly decreased with *p* < 0.0001. The high content of ethanol produces a negative charge on the polar heads of the phospholipids resulting in electrostatic repulsion. This repulsion not only prevents the aggregation of nanoparticles and promotes the stability properties [69] but also the negative charge on vesicles is responsible for better penetration through the skin layers [71]. On the other hand, a non-significant (*p* = 0.2601) increase in the ZP was observed by increasing the surfactant concentration [63]. The reason behind this increment was that the increased non-ionic surfactant got adsorbed on the particle surface and caused the overall reduction in charge on the surface of particle [72]. The increased concentration of lipid resulted in the reduction in ZP owing to the increased concentration of negatively charged components of the phospholipid (*p* = 0.0762) [67].

### 3.5. Optimization of nitazoxanide-loaded transethosomal gel

After loading the different concentrations of NTZ in optimized blank TES, the physical unstability in the form of precipitation and reduced %EE was observed which might be owing to the partial lipid bilayer solubilization in high ethanol content, resulting in leaky vesicles leading to the loss of drug and thus reduced %EE [69]. Moreover, at a high concentration of ethanol when the drug was loaded to the blank TES, the loss of drug from vesicles was observed because of the disturbance caused by the drug in steric stabilization of vesicles imparted by ethanol [68]. Thus, the ethanol concentration was reduced up to 30% without disturbing other components and evaluation of PS, PDI, ZP and %EE was done. By reducing the ethanol concentration, a significant increase in %EE was observed because at a higher concentration of ethanol, drug leaches out of vesicles because of solubilization of the drug in ethanol as well as increased permeation of vesicle membranes owing to high fluidity imparted by ethanol [68]. Optimization of NTZ-TES formulations in terms of PS, PDI, ZP and %EE is shown in table 2.

**Table 2.** Optimization of the drug-loaded-TES. (All the components of the above mentioned formulations were taken in 10 ml. All values here represent mean ± s.d. (*n* = 3). PS, particle size; PDI, polydispersity index; ZP, zeta potential.)

formulation code	lipid (mg)	surfactant (mg)	ethanol (%)	NTZ (mg)	PS (nm)	PDI	ZP (mV)	EE (%)
TES-NTZ-1	91.8	10	40	5	127.5 ± 7.05	0.168 ± 0.032	−20.2 ± 0.62	69.74 ± 0.77
TES-NTZ-2	91.8	10	40	4	138.2 ± 7.13	0.100 ± 0.009	−18.0 ± 0.80	62.49 ± 0.86
TES-NTZ-3	91.8	10	40	3	142.8 ± 6.29	0.077 ± 0.011	−16.9 ± 0.85	44.19 ± 0.78
TES-NTZ-4	91.8	10	30	5	176.6 ± 9.95	0.093 ± 0.017	−26.4 ± 2.88	86.03 ± 0.15
TES-NTZ-5	91.8	10	30	4	188.4 ± 8.35	0.094 ± 0.022	−29.9 ± 1.21	90.49 ± 0.52

**Figure 3.**
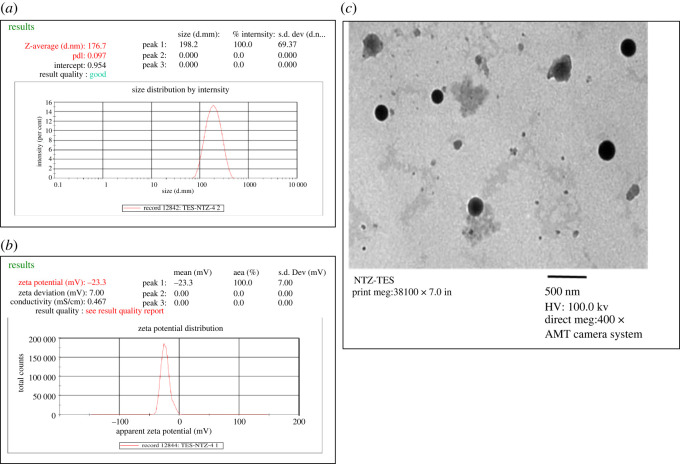
Particle properties of NTZ-TES, (*a*) PS; (*b*) ZP; (*c*) TEM, 4000X.

TEM analysis of NTZ-TES showed that the particles were spherical in shape, sealed and uniformly distributed with a smooth outer bilayer indicating lower PDI [73]. Moreover, the PS indicated by TEM analysis was in accordance with the Zetasizer results. However, some particles with irregular spherical shape were also observed which might be owing to the disturbance of the lipid bilayer of vesicles caused by ethanol and/or surfactant [74]. The results of PS, ZP, PDI and TEM are shown in figure 3.

**Figure 4.**
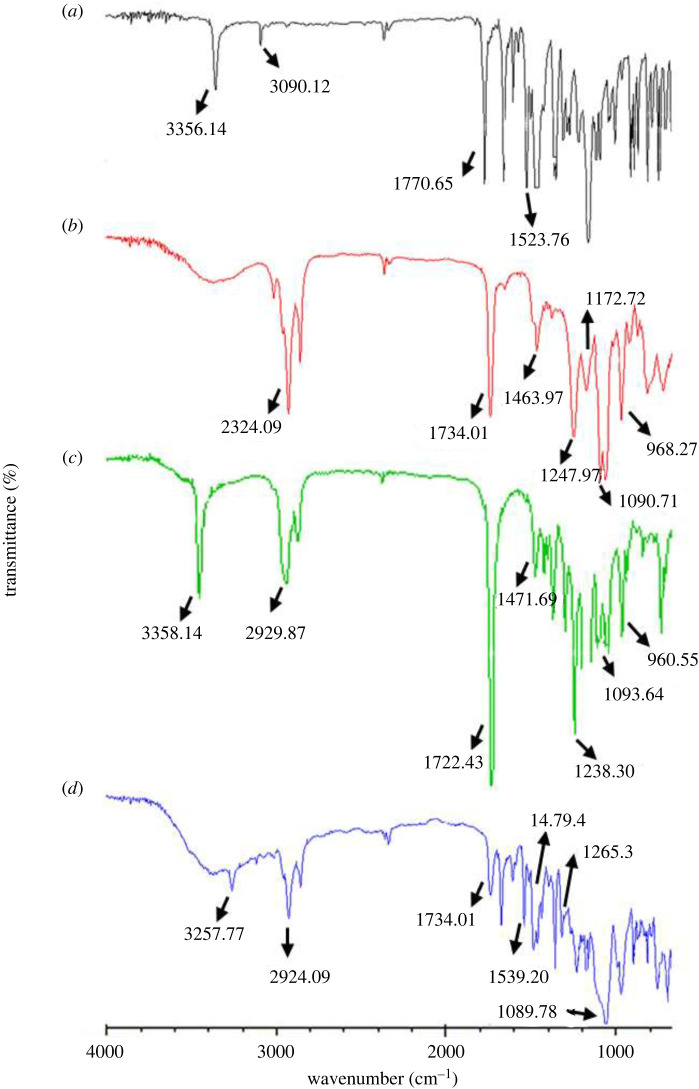
FTIR spectra of (*a*) NTZ, (*b*) PLG 90, (*c*) PM, and (*d*) NTZ-TES.

The FTIR spectrum of NTZ (figure 4*a*) indicated a characteristic peak of N-H stretching at 3356.14 cm^−1^ and aromatic C-H stretching at 3090.12 cm^−1^. The other characteristic bands at 1770.65 cm^−1^ and 1523.76 cm^−1^ are indicative of the C = O and N = O stretching, respectively [75]. On the other hand in the FTIR spectrum of PL90G shown in figure 4*b*, the absorbance band at 2924.09 cm^−1^ indicated the O-H stretching, while the characteristic peak at 1172.72–1247.94, 968.27 cm^−1^ and 1090.71 showed the P=O, N(CH_3_)_3_ and P-O-C stretching, respectively [76]. The absorbance band at 1734.01 cm^−1^ indicates the C=O stretching of the phospholipid [77]. Moreover, in the FTIR spectra of PM and NTZ-TES (figure 4*c*,*d*), all the characteristic peaks of NTZ and PL90G were intact indicating no interaction between drug and excipients. The only change in intensity of the characteristic peak of PL90G at 1734.01 cm^−1^ (C=O) in NTZ-TES spectra was owing to the formation of H-bonds between the OH-groups and carbonyl groups of the phospholipid. Furthermore, this intensity change in peak is the indicator of change in the relative number of the free and hydrogen-bonded C=O groups. The band at 1463.97 cm^−1^ is an indicator of the -CH_2_- group of the fatty acid chains of the lipid and the span [77]. The shift of the absorbance bands of P-O-C and P=O at 1090.71 cm^−1^ and 1247.94 cm^−1^ to 1089.78 cm^−1^ and 1265.3 cm^−1^ in NTZ-TES spectrum was the indicator of the interference caused at the polar heads of the PL90G in the TES [76].

### 3.6. Physico-chemical and rheological study of nitazoxanide-loaded transethosomal gel

On physical inspection, the NTZ-TEG was clear opaque, homogeneous in texture, and pale yellow in colour owing to NTZ. The pH of the NTZ-TEG was 5.02 ± 0.11 (table 3), which is within the range of normal pH range reported for skin i.e. 4–6 [78]. In a rheological study, by increasing the shear rate, the viscosity of the gel was decreased, indicating the shear thinning behaviour of gel which is the desirable characteristic for topical application of gels [79,80].

**Table 3.** Characterization of blank gel and NTZ-TEG.

parameters	NTZ-TEG	blank gel
pH	5.02 ± 0.11	5.1 ± 0.12
appearance	clear opaque gel	clear transparent gel
homogeneity	uniform	uniform
colour	pale yellow	colourless
viscosity (cP)	32 000 ± 15	31 160 ± 20

### 3.7. Measurement of spreadability of nitazoxanide-loaded transethosomal gel

In order to evenly and homogeneously apply the gel on skin, it must have good spreadability [48]. The spreadability measurements were taken in triplicate and % spreadability of NTZ-TEG, and blank gel was obtained as 280 **±** 26.46% and 313.33 **±** 18.92%, respectively, which indicated the good spreadability of the gel [79].

### 3.8. *In vitro* release and kinetic drug release study

**Figure 5.**
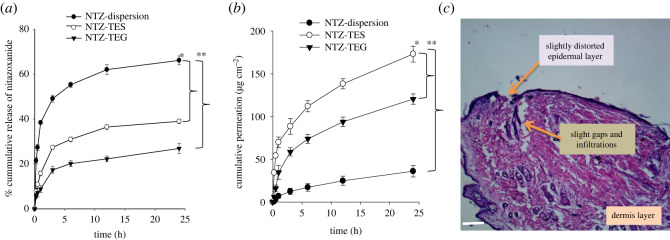
(*a*) *In vitro* release profile of NTZ-dispersion, NTZ-TES and NTZ-TEG at pH 5.5, (*b*) *ex vivo* permeation study of NTZ-dispersion, NTZ-TES and NTZ-TEG in PBS 7.4 pH, and (*c*) histopathological study of skin treated with plain NTZ-dispersion indicating the slightly distorted epidermal layer and infiltrations. Bar length represents 50 µm scale.

The *in vitro* release of NTZ from NTZ-dispersion, NTZ-TES and NTZ-TEG was analysed at pH 5.5 (endosomal pH) [81]. The release of NTZ from NTZ-dispersion was rapid and almost 56% drug was released after 24 h of the study (figure 5*a*), while in the case of the NTZ-TES and NTZ-TEG, the release of NTZ was low and prolonged in comparison with the pure drug dispersion. After 3 h, approximately 30% drug was released from NTZ-TES and 15% from NTZ-TEG, while after 24 h of the study almost 40% drug release was observed in case of NTZ-TES and 22% drug was released from NTZ-TEG**,** indicating a slower and prolonged release profile. In the case of NTZ-TEG, the drug release rate was much lower than the NTZ-TES, because the drug has to cross two barriers, i.e. first to release from the TES and second from the chitosan gel matrix. The data of drug release was subjected to different release kinetic models in order to understand the mechanism/model which was being followed for drug release from TES and gel (electronic supplementary material, table S3). The Korsmeyer-Peppas model showed highest *R*^2^ values for both the NTZ-TES and NTZ-TEG. Moreover, the values of diffusion coefficient (*n*) (electronic supplementary material, table S4) for both NTZ-TES and NTZ-TEG were less than 0.5 indicating that the release followed the quasi-Fickian diffusion mechanism [82].

### 3.9. *Ex vivo* skin permeation study and drug deposition study

An *ex vivo* permeation experiment was conducted for NTZ-dispersion, NTZ-TES and NTZ-TEG at pH 7.4 using the Franz diffusion cell. The per unit time cumulative concentration of NTZ permeated was estimated by plotting the cumulative amount (µg cm^−2^) on the *y*-axis and time (h) on the *x*-axis as shown in figure 5*b*. The penetration rate of NTZ-TES was significantly higher than NTZ-TEG and NTZ-dispersion (electronic supplementary material, table S5). Within 1 h, 69.84 µg cm^−2^ of NTZ-TES was permeated while 34.69 µg cm^−2^ of NTZ was permeated from NTZ-TEG. On the other hand, only 6.9 µg cm^−2^ of NTZ was permeated from plain NTZ-dispersion within 1 h, which was further increased with the passage of time owing to the mild skin irritation and disruption caused by the pure NTZ-dispersion, also confirmed by the histopathological study of skin as shown in figure 5*c*. The permeation of drug from the NTZ-TEG was slower in comparison with the NTZ-TES. The permeability coefficient and flux of NTZ-TES were approximately seven and five times higher than the NTZ-dispersion, while for the NTZ-TEG flux and Kp were approximately three and five times higher than the plain drug dispersion, respectively. Thus, the overall results proved that NTZ-TES have high skin penetrating potential in comparison with the pure drug. This high penetration rate of the NTZ-TES might be owing to its ultra-deformable nature and increased fluidity of membrane imparted by high ethanol content. TES have both the edge activator-like TES and a high ethanol percentage just like ethosomes, thus it has properties of both the vesicles and also shares the mechanism of penetration of both transferosomes and ethosomes [83].

**Figure 6.**
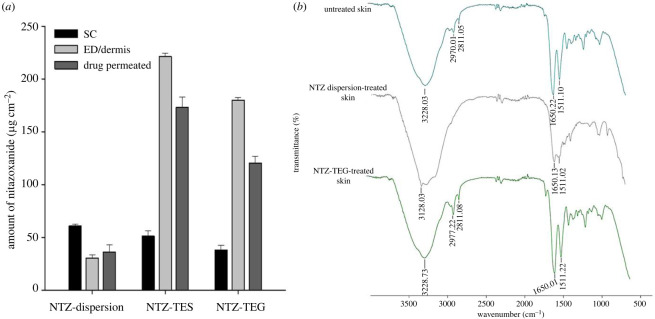
(*a*) The amount of NTZ permeated and retained in different skin layers, i.e. SC, epidermal (ED)/dermal layer after treatment with NTZ-dispersion, NTZ-TES and NTZ-TEG; and (*b*) FTIR spectra of rat skin after treatment with NTZ-TEG and NTZ-dispersion.

After 24 h of skin permeation study, the skin was removed, and a skin deposition study was performed. The results of the skin deposition study showed a higher retention of NTZ-TES in the dermal layer of the skin which was owing to its highly deformable nature along with promoting effect of ethanol on penetration. The ethanol in TES when applied to skin increases the fluidity of lipid bilayers of SC, thus enhances the deeper skin penetration of TES [84]. As shown in figure 6*a*, the NTZ-TES and NTZ-TEG showed higher drug retention in skin layers as compared to the NTZ-dispersion, and the deposition of NTZ was higher in the dermal layer of skin in comparison with the SC layer. The observed results proved that by using TES deeper skin layer permeation can be achieved, and the deposition of TES in the dermal layer provides the prolonged supply of NTZ for enhanced therapeutic effect and reduced systemic adverse effects [84].

### 3.10. Evaluation of skin after treatment

The FTIR of the skin was performed in order to assess the structural changes after treatment with NTZ-TEG and NTZ-dispersion. FTIR analysis of untreated, NTZ-TEG-treated and NTZ-dispersion-treated rat skin was performed. The peaks at 3228.03 cm^−1^ and 3228.73 cm^−1^ in the untreated and NTZ-TEG-treated skin, respectively, confirmed that the hydrocarbons of the skin lipid bilayer were intact, and no damage was caused by the NTZ-TEG after their topical application. However, in case of NTZ-dispersion-treated skin, this peak was shifted to 3428.03 cm^−1^ indicating disturbance in lipid bilayer of the skin figure 6*b*. Moreover, absorbance bands at 2970.01 cm^−1^ and 2811.05 cm^−1^ in case of untreated skin indicated the symmetric and asymmetric vibrations of CH_2_ bonds of the hydrocarbons of lipids. However, in case of NTZ-TEG, the intensity of peaks at 2977.22 cm^−1^ and 2811.08 cm^−1^ was reduced in comparison with the untreated skin, indicating the slight extraction of lipids from the SC by TES. On the other hand, the skin treated with NTZ-dispersion showed no peaks of symmetric and asymmetric CH_2_ bonds of lipids, indicating the complete extraction of lipids of the SC layer of skin. Untreated skin showed peaks at 1650.22 and 1511.10 cm^−1^ which indicates the amide bonds of proteins of the SC, i.e. C=O stretching and C-N stretching, respectively. In case of NTZ-TEG, the peaks of the amide bonds i.e. 1650.01 and 1511.22 cm^−1^ were intact indicating no change in protein structure, while in case of NTZ-dispersion, a significant reduction in intensity of peaks at 1650.13 and 1511.02 cm^−1^ was observed which indicated the interaction of NTZ with the proteins of the skin [50,85]. The results of FTIR spectra of skin proved that the NTZ-TEG caused no alteration in skin structure in comparison with the pure NTZ-dispersion which caused the alterations in lipids as well as protein structures of skin layers. Thus, the optimized TES formulation crossed the skin because of its ultra-deformable characteristic without damaging the skin organization.

### 3.11. *In vivo* histopathology and skin irritation study

**Figure 7.**
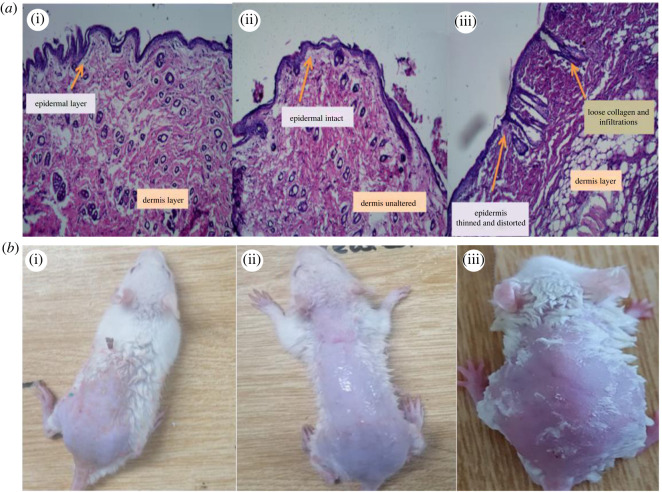
(*a*) Histopathological images of (i) untreated skin; (ii) NTZ-TEG-treated skin; and (iii) 0.8% formalin-treated skin; (*b*) Images of rat skin treated with (i) untreated; (ii) NTZ-TEG; and (iii) 0.8% formalin. Bar length represents 50 µm scale.

Skin irritation limits the use and patient compliance in case of topical formulations, thus the topical gel must be non-irritant and safe to apply [86,87]. A skin irritation study was conducted to assess potential irritation caused by the formulation and results were further confirmed by histopathological study. The scoring was done according to the Draize scoring method (electronic supplementary material, table S6) [53]. The results of the *in vivo* skin irritation study showed that the PDII of NTZ-TEG was 0.4 in comparison with a standard irritant, i.e. 0.8% formalin indicated a PDII of 4 (electronic supplementary material, table S7). The obtained score of less than 2 indicates the non-irritant and good safety profile of the NTZ-TEG in comparison with the standard skin irritant. The results of histopathological study indicated that the epidermal layer was intact and no signs of infiltration or loose collagen fibres were observed in case of skin treated with NTZ-TEG in comparison with the standard skin irritant. The histopathological results of NTZ-TEG-treated skin were the same as the normal or untreated skin histology as shown in figure 7.

### 3.12. *In vitro* leishmanicidal activity against promastigotes

The *in vitro* anti-leishmanial assay against the promastigotes indicated a mean percentage inhibition of 51.99 ± 1.29% in case of NTZ-TES which was significantly higher in comparison with the NTZ-dispersion. The IC_50_ values of 13.4 ± 1.76 and 6.9 ± 0.29 µg ml^−1^ were obtained for NTZ-dispersion and NTZ-TES, respectively (table 4). The significantly reduced IC_50_ value for NTZ-TES in comparison with the NTZ- dispersion indicated the better and enhanced efficacy of NTZ-TES against the promastigotes of *L. tropica*.

**Table 4.** Mean percentage inhibition and IC_50_ values of NTZ-TES and NTZ-dispersion. (All values here represent mean ± s.d. (*n* = 3).)

formulation	inhibition (%)	IC_50_ (µg ml^−1^)
NTZ-dispersion	19.74 ± 1.19	13.4 ± 1.76
NTZ-TES	51.99 ± 1.29	6.9 ± 0.29

### 3.13. Cell uptake study

The macrophage uptake of NTZ was quantified by incubating the NTZ-dispersion and NTZ-TES with previously harvested macrophage cells. It was observed that 4.08 ± 0.41 µg (8%) NTZ was assimilated by the macrophages treated with plain drug dispersion while in the case of NTZ-TES, 12.8 ± 0.32 µg (25.6%) NTZ was recovered from the lysis of 2 × 10^4^ macrophage cells. The internalization of nano-TES were significantly higher than the plain drug dispersion which might be because of the nano size of the vesicles, i.e. less than 200 nm. This enhanced internalization of NTZ-TES could improve the efficacy of NTZ against leishmanial parasites as well as possibly acting as a drug depot releasing drug in the close proximity of the parasites residing in the macrophages [88,89].

### 3.14. Stability study

Stability study was performed for the NTZ-TES for a period of four months at room temperature and refrigerating temperature. The nanoparticles were characterized with regard to physical stability, PS, PDI and ZP. The results indicated non-significant variation in ZP, PS and PDI at temperature of 4 ± 2°C as shown in table 5. While at a temperature of 30 ± 2°C, an increase in PS, PDI and ZP was observed as shown in table 6. Thus, the suitable storage temperature for NTZ-TES was proved to be 4 ± 2°C.

**Table 5.** Stability study of NTZ-TES at 4 ± 2°C. (All values represent mean ± s.d. (*n* = 3). PS, particle size; PDI, polydispersity index; ZP, zeta potential; mV, millivolt; nm, nanometer.)

time period	PS (nm)	PDI	ZP (mV)	physical stability
0 days	164.17 ± 2.56	0.099 ± 0.012	−23.11 ± 1.25	stable
one month	164.33 ± 5.01	0.101 ± 0.016	−22.50 ± 1.70	stable
two months	164.53 ± 0.95	0.108 ± 0.027	−22.41 ± 1.28	stable
three months	165.23 ± 1.43	0.113 ± 0.012	−22.06 ± 1.01	stable
four months	165.5 ± 0.57	0.121 ± 0.021	−21.83 ± 1.02	stable

**Table 6.** Stability study of NTZ-TES at 30 ± 2°C. (All values represent mean ± s.d. (*n* = 3). PS, particle size; PDI, polydispersity index; ZP, zeta potential; mV, millivolt; nm, nanometer.)

time period	PS (nm)	PDI	ZP (mV)	physical stability
0 days	164.17 ± 2.56	0.099 ± 0.012	−23.11 ± 1.25	stable
one month	173.8 ± 2.59	0.119 ± 0.019	−19.50 ± 1.70	stable
two months	181 ± 4.33	0.089 ± 0.016	−7.04 ± 0.79	stable
three months	184.37 ± 11.58	0.144 ± 0.021	−4.69 ± 0.60	stable
four months	205.4 ± 0.14	0.106 ± 0.009	−4.66 ± 0.44	stable

## 4. Conclusion

The NTZ-TES were successfully prepared and optimized in terms of PS, PDI, ZP, TEM and FTIR. The optimized NTZ-TES were nano-sized, having low PDI, spherical in shape, and no interaction was found between the excipients of TES. The characterization of NTZ-TEG indicated uniform consistency, optimal pH for skin application, good spreadability, desirable viscosity and shear thinning nature of the gel which were the desired characteristics for topical gel application. The *in vitro* release study of NTZ-TES and NTZ-TEG showed a prolonged release behaviour and mechanism of release was diffusion controlled. The *ex vivo* skin permeation study of NTZ-TES and NTZ-TEG indicated a higher permeability of TES to deeper skin layers and enhanced retention in the dermal layer of skin for local effect. The *in vivo* skin irritation and histopathology study of NTZ-TEG showed a safe and non-irritant behaviour of NTZ-TEG. The higher percentage inhibition and lower IC_50_ value for NTZ-TES against promastigotes of *L. tropica* indicated its efficacy against CL. The macrophage targeting performance of NTZ-TES was confirmed by the quantitative cellular uptake study. In the light of overall results, it is concluded that the NTZ-TEG can be effectively used for the topical delivery of NTZ in CL.

## Data Availability

The data are available from the Dryad Digital Repository: https://doi.org/10.5061/dryad.cfxpnvx7d [90]. Data are also provided in the electronic supplementary material [91].
